# Infodemic as a structural barrier to sexual and reproductive health service utilization in Africa: a rapid critical narrative review

**DOI:** 10.3389/frph.2026.1776832

**Published:** 2026-05-07

**Authors:** Bahizire Riziki Richard, Aganze Mitima Synthia

**Affiliations:** 1 Unity of Epidemiology, Sub Unity of Infodemic Management, School of Public Health, University of Lubumbashi, Lubumbashi, Democratic Republic of Congo; 2 Department of Public Health, Higher Institute of Medical Techniques of Nyangezi, Nyangezi, Democratic Republic of Congo; 3 Department of Public Health, University of Parakou, Parakou, Benin

**Keywords:** Africa, health literacy, health systems, infodemic, misinformation, sexual and reproductive health, syndemic theory

## Abstract

**Background:**

Sexual and reproductive health (SRH) services are crucial for public health, gender equality, and sustainable development in Africa. However, the use of these services has been increasingly hindered by infodemic, characterized by an overwhelming amount of information, whether accurate or not, that complicates access to trustworthy guidance. Although infodemic is often seen mainly as issues of digital misinformation, their wider structural, social, and epistemic aspects within the African context have not been thoroughly examined in the literature.

**Methods:**

This study conducted a rapid critical narrative review of literature published from 2020 to 2025. From a total of 38 sources, 21 articles specifically focusing on SRH and information environments in Africa were selected for a detailed interpretive synthesis. To ensure representation across the continent, the review incorporates evidence from 12 countries, including those in Western (Nigeria, Ghana), Central (Gabon, Angola, DRC), Eastern (Kenya, Ethiopia, Rwanda, Burundi), Northern (Egypt, Morocco), Southern (South Africa), and Insular Africa (Madagascar). Findings: The synthesis reveals that SRH-related infodemic serve as structural barrier through four interconnected mechanisms: (1) scientific uncertainty and information gaps during health crises, (2) socially ingrained myths stemming from historical institutional distrust, (3) the “digital-to-oral” translation pathway, and (4) gender-specific disparities in health literacy.

**Conclusion:**

Infodemic act as structural barriers to SRH services, rather than merely isolated communication breakdowns. Enhancing SRH systems necessitates the development of robust communication ecosystems that bridge the digital divide, involve community leaders, and honor local historical contexts across Africa's varied landscapes.

## 1 Introduction

Sexual and reproductive health (SRH) services, including family planning, maternal care, and HIV/STI prevention, are fundamental human rights across the African continent. Despite significant progress, the utilization of these services remains inequitable, influenced by deeply rooted structural inequities, restrictive gender norms, and health system constraints (1, 2). Gender norms are critical contextual factors that often limit women's autonomy in making health-related decisions. In many African settings, traditional roles restrict access to health information, directly impacting individuals' ability to seek timely care (3, 4).

Beyond supply side constraints, misinformation and uncertainty, which are amplified by social dynamics, have emerged as major hurdles. An infodemic, an overabundance of accurate and inaccurate information, undermines individuals ability to identify reliable health sources (5). In contexts where formal information is fragmented, confusion and hesitancy ensue. These dynamics are particularly acute in settings characterized by historical mistrust of health systems and reliance on informal communication networks (6, 7).

Despite the role of information environments, the literature has predominantly focused on healthcare availability, overlooking how information environments, as structural barriers, influence decision-making. There is a clear gap in understanding the syndemic nature of infodemic, how information crises interact with existing social and health vulnerabilities on a continental scale (8–10). By analyzing evidence from Western, Central, Eastern, Northern, Southern, and Insular Africa, this review explores the role of information environments in SRH utilization and proposes strategies for building resilient communication ecosystems.

## 2 Methods

### 2.1 Study design

To capture the complexity of how information environments shape health behaviors, we adopted a rapid critical narrative review methodology. This approach was chosen specifically to allow for an integrative and context-sensitive interpretation of diverse evidence. In emerging and interdisciplinary fields such as “infodemic studies,” valuable knowledge is often scattered across empirical research, policy analyses, and grey literature. Rather than a simple statistical aggregation, our design prioritizes conceptual integration and the identification of the underlying mechanisms that explain why and how information dynamics act as barriers to care (11–14).

### 2.2 Conceptual orientation

To provide a deeper analytical meaning to our findings, we examined four complementary conceptual lenses. First, we applied the WHO infodemic management framework to understand information ecosystems. This was paired with syndemic theory, which allowed us to examine how social and health crises reinforce one another. We also drew on health system resilience perspectives—focusing on trust and governance—and gender- and rights-based approaches to highlight power imbalances and patient autonomy.

Within this framework, we move beyond viewing infodemic as mere “individual cognitive gaps.” Instead, we conceptualize them as structural and relational phenomena deeply rooted in the historical, political, and institutional contexts of African societies.

### 2.3 Sources and selection process

Our search focused on literature published between 2020 and 2025. This timeframe was a deliberate choice, as it allowed us to capture the evolution of information dynamics following the formalization of the “infodemic” concept by the WHO at the start of the COVID-19 pandemic.

We explored PubMed/MEDLINE, Scopus, Web of Science, and Google Scholar using targeted keyword combinations such as: (“Infodemic” OR “Misinformation” OR “Rumors”) AND (“Sexual and Reproductive Health” OR “Family Planning”) AND (“Africa”).

From an initial pool of 38 sources, we rigorously selected 21 articles (2–4, 6–10, 22–30, 33–38) for a full interpretive synthesis. These specific papers were chosen for their direct focus on African SRH contexts, representing a diverse geographic spread of 12 countries across the continent's five major regions and its islands (Madagascar). The remaining 17 sources (1, 5, 11–21, 31, 32) were used to establish the theoretical and methodological foundations of our study.

### 2.4 Data analysis

Rather than a mere compilation of data points, we conducted an interpretive thematic synthesis. This process involved a three-step journey: meticulous line-by-line coding of findings from the included papers, development of descriptive themes, and finally, generation of analytical themes that connect infodemic dynamics to structural SRH barriers. To ensure the reliability of this interpretation and mitigate individual bias, all themes were cross-checked and refined through collaborative peer discussions within our research team.

## 3 Key findings : infodemic dynamics and sexual and reproductive health service utilization

The evidence synthesized from the selected literature reveals distinct regional patterns in how misinformation impacts health outcomes across the continent (Table 1).

**Table 1:** Continental distribution of the 21 synthesized articles.

Region	Key Countries	SRH Focus	Critical Infodemic Mechanism	Key Sources
Western	Nigeria, Ghana, Senegal	Contraception, Youth	WhatsApp rumors & Religious gatekeeping	(24, 33–35)
Central	Gabon, Angola, DRC	HPV Vaccination, Maternal Health	Information voids & “Sterilization” narratives	(7, 35, 36)
Eastern	Kenya, Ethiopia, Rwanda, Burundi	Adolescents, Abortion care	Cultural myths & Digital-to-Oral translation	(8, 25, 31, 35)
Northern	Morocco, Egypt	Maternal Health, Norms	Gender inequities & Digital access barriers	(3, 9, 34, 38)
Southern	South Africa	HIV/AIDS, Refugee Women	Stigmatization & Institutional mistrust	(2, 26–28, 30)
Insular	Madagascar	Prenatal Care	Systemic fear linked to epidemic outbreaks	(7, 37)

### 3.1 Exploitation of “informational voids” and institutional distrust

A significant finding pertains to the emergence of the infodemic during service disruptions. In the Democratic Republic of Congo, Gabon, and Madagascar, health crises such as Ebola and COVID-19 led to “institutional silences,” which were perceived as admissions of powerlessness or concealment. These “voids” were not only filled by rumors but also by a reinterpretation of fear. In the collective imagination, hospitals became sites for the “capture” of data or bodies, resulting in a drastic decline in the utilization of SRH services, which were perceived as vectors of non-consensual experimentation (7, 25, 37).

### 3.2 Resilience of myths and “epistemic violence”

In Kenya, Ethiopia, and Nigeria, rumors of forced sterilization via vaccines (HPV) or contraception are not mere misunderstandings but manifestations of traumatic memory. The study reveals that these narratives are responses to “epistemic violence”: the perception that SRH programs are imposed by external agendas without consideration for local realities. “Fact-checking” often fails because it is perceived as a renewed attempt at intellectual domination rather than medical assistance (8, 26, 28, 35).

### 3.3 The political economy of platforms: the “zero-rating trap”

A crucial mechanism identified in Nigeria and Ghana is the structural role of digital infrastructures. “Zero-rating” programs (which provide free access to Facebook/WhatsApp but require paid data for official health sites) create an access asymmetry. Disinformation circulates “freely” and without limit, while scientific truth is “taxed” by the cost of mobile data. This mechanism structurally subsidizes the circulation of SRH myths, trapping low-income populations in a disinformation bubble that is lucrative for platforms but lethal for public health (32, 34).

### 3.4 Digital-to-Oral translation and the 2.0 word-of-mouth (*radio-trottoir*)

In Rwanda, Burundi, and South Africa, we identified a transcoding process. Misinformation originating on TikTok or Twitter is “translated” by local opinion leaders into oral narratives adapted to cultural codes. This “Radio-Trottoir 2.0” (street radio) allows digital disinformation to bridge the digital divide and reach the most remote rural areas. For these populations, oral rumors carried by a neighbor hold more epistemic weight than any official radio campaign (2, 23, 24).

### 3.5 Epistemic gatekeepers: religious and traditional leaders

In Senegal, Egypt, and Ethiopia, the infodemic is arbitrated by religious authorities acting as filters. When SRH messaging (regarding reproductive autonomy or safe abortion) conflicts with dogma, these leaders do not merely reject the information; they reconfigure it as a “moral threat.” Factual information is thus discarded in favor of a discourse centered on protecting cultural identity, rendering SRH services socially inaccessible (35, 37, 38).

### 3.6 The intersectionality of vulnerability: refugees and minorities

In South Africa and Kenya, the infodemic specifically targets marginalized groups. Refugee women and LGBTQ+ populations are subjected to “stigmatizing” disinformation that portrays them as disease vectors or targets for state repression via health centers. For these groups, the infodemic transforms SRH centers into potential danger zones (of deportation or arrest), leading to the total invisibilization of their health needs (2, 30, 33).

#### 3.2.7 Gender inequities and “information poverty”

In Morocco and Egypt, gender norms restrict women's access to smartphones or independent data plans. This informational dependency on male family members creates a structural barrier: women can often only verify a rumor through the lens of their social circle, which is frequently influenced by prejudice. The infodemic exploits this power asymmetry to restrict women's reproductive autonomy (3, 9, 34).

## 4 Discussion

### 4.1 The syndemic nature of the SRH infodemic: beyond simple rumors

The findings of this review suggest that the infodemic within the African SRH landscape functions as a formidable structural barrier, eroding the very foundation of health-seeking behavior: trust (7, 22). By interpreting these results through the lens of syndemic theory, it becomes evident that information crises do not emerge in a vacuum (17, 18). Instead, they coalesce with pre-existing social vulnerabilities, historical grievances, and gender inequities to create a self-reinforcing cycle of exclusion (2, 35). In contexts such as South Africa and the DRC, the intersection of acute health crises (e.g., Ebola or COVID-19) with a deep-seated “trust deficit” toward state institutions has transformed public health guidelines into perceived threats to bodily autonomy (7, 30).

### 4.2 The weight of the past: historical grievances and “protective truths”

A critical insight from this synthesis is the resilience of conspiracy narratives in countries like Kenya, Ethiopia, and Nigeria (8, 26, 28). Evidence confirms that “fact-checking” is often an insufficient clinical remedy for what is essentially a socio-political ailment (20, 21). When misinformation resonates with historical distrust, frequently rooted in colonial-era medical abuses or unethical interventions, it ceases to be a mere “error” and becomes a protective social narrative (6, 27). Building epistemic trust therefore requires health systems to move beyond technical debunking toward a relational approach that acknowledges these historical traumas as legitimate determinants of contemporary health behaviors (1, 20).

### 4.3 The political economy of the click: the “digital-to-oral” pathway and the zero-rating trap

The “digital-to-oral” translation pathway identified in Rwanda, Burundi, and Ghana highlights a significant gap in global infodemic management strategies (23, 24). While international protocols focus predominantly on social media algorithms (5, 16), the African context reveals that the most potent rumors are those that migrate “from the screen to the street” (23, 35). Furthermore, the political economy of digital platforms plays a silent but decisive role: “Zero-rating” programs, where social media access is free while external fact-checking websites require paid data, create an asymmetrical information economy (34). This effectively subsidizes the spread of myths while “taxing” the pursuit of scientific truth, a structural barrier that heavily impacts rural and low-income populations (34, 38).

### 4.4 Mediators and sentinels: religious and traditional leaders as epistemic gatekeepers

Analysis across Senegal, Ethiopia, and Egypt reveals that infodemics are often mediated through religious and traditional authority figures (35, 37). These actors are not merely information consumers; they function as epistemic gatekeepers (35). When official SRH guidance clashes with theological interpretations, particularly regarding adolescent autonomy or family planning, religious networks can inadvertently amplify moralized misinformation (32, 35). Achieving resilience in SRH utilization requires moving beyond clinical outreach toward a “cultural diplomacy” that aligns public health goals with local value systems (1, 19).

### 4.5 Gendered environments and the invisibility of marginalized groups

The gendered nature of information environments in Morocco and Egypt underscores that SRH autonomy is inextricably linked to informational autonomy (3, 9). When structural gender norms dictate who can access digital tools or participate in public discourse, the information environment itself becomes an instrument of exclusion (9, 34). Additionally, infodemic disproportionately impact highly vulnerable groups, such as refugees in South Africa or Kenya, who face targeted disinformation that fuels stigma (2, 33). For these groups, a rumor is not just a barrier to a clinic; it is a direct threat to their physical safety, often leading to a total “invisibilization” of their health needs (2, 30).

### 4.6 Towards health system resilience and information sovereignty

To build truly resilient health systems, African policymakers must shift focus from “supply-side” metrics toward the “trust-side” of the ecosystem (1). Resilience should not be measured only by the distribution of commodities, but by the capacity to maintain a respectful, transparent, and bidirectional dialogue with the community (1, 16). Finally, there is a clear need for Information Sovereignty: empowering African communities to co-produce health narratives in local languages (21, 34). True resilience is achieved when communities possess the tools to filter information based on both scientific accuracy and cultural relevance (21, 31).

## 6 Conclusion

This systematic review demonstrates that the infodemic within the African landscape is not merely a technical challenge of content moderation, but a profound structural determinant of Sexual and Reproductive Health (SRH) service utilization. By synthesizing evidence from 12 countries across Western, Central, Eastern, Northern, Southern, and Insular Africa, we have shown that information crises are not isolated communication failures. Instead, they are deeply embedded in historical grievances, gender inequities, and the political economy of digital platforms.

Our analysis highlights four critical mechanisms, institutional “informational voids,” historical conspiracy narratives (epistemic violence), the “digital-to-oral” translation pathway, and gendered information gaps, that collectively undermine epistemic trust in health systems. We argue that addressing these dynamics requires a fundamental paradigm shift: moving beyond simple “fact-checking” toward building resilient and sovereign communication ecosystems.

To achieve equitable SRH outcomes and build health system resilience, African policy-makers and practitioners must prioritize the following strategic interventions:

1. Integrate Community-Based Oral Networks: Formal health communication must move “from the screen to the street” by engaging local oral circuits to counter the potent spread of “Radio-Trottoir 2.0” and street-level rumors.
2. Foster Cultural and Theological Diplomacy: Rather than viewing dogma as a barrier, health systems must engage religious and traditional leaders as epistemic gatekeepers and active co-producers of health information that aligns with local value systems.
3. Address the Digital Inequity Gap: Structural reform is needed to dismantle the “zero-rating” trap, ensuring that verified scientific truth is as accessible and “cost-free” as the misinformation currently subsidized by platform architectures.
4. Promote Digital Health Sovereignty: Empowering local communities to generate health narratives in their own languages and cultural contexts ensures that SRH messaging is not perceived as an external imposition but as a communal asset.

Ultimately, the resilience of African health systems depends on the ability to maintain a transparent, respectful, and bidirectional dialogue with the community. In the face of future pandemics or information crises, the “trust-side” of the health ecosystem will be just as vital as the “supply-side” in ensuring that no one is discouraged from exercising their fundamental right to sexual and reproductive health.
